# Influence of Hydration Status on Changes in Plasma Cortisol, Leukocytes, and Antigen-Stimulated Cytokine Production by Whole Blood Culture following Prolonged Exercise

**DOI:** 10.1155/2014/561401

**Published:** 2014-03-12

**Authors:** Ida S. Svendsen, Sophie C. Killer, Michael Gleeson

**Affiliations:** School of Sport, Exercise and Health Sciences, Loughborough University, Ashby Road, Loughborough, Leicestershire LE11 3TU, UK

## Abstract

Elevated antigen-stimulated anti-inflammatory cytokine production appears to be a risk factor for upper respiratory tract illness in athletes. The purpose of this study was to determine the effects of prolonged exercise and hydration on antigen-stimulated cytokine production. Twelve healthy males cycled for 120 min at 60% $\dot{V}{\text{O}}_{2\max}$ on two occasions, either euhydrated or moderately hypohydrated (induced by fluid restriction for 24 h). Blood samples were collected before and after exercise and following 2 h recovery for determination of cell counts, plasma cortisol, and *in vitro* antigen-stimulated cytokine production by whole blood culture. Fluid restriction resulted in mean body mass loss of 1.3% and 3.9% before and after exercise, respectively. Exercise elicited a significant leukocytosis and elevated plasma cortisol, with no differences between trials. IL-6 production was significantly reduced 2 h postexercise (*P* < 0.05), while IL-10 production was elevated postexercise (*P* < 0.05). IFN-**γ** and IL-2 production tended to decrease postexercise. No significant effect of hydration status was observed for the measured variables. Prolonged exercise appears to result in augmented anti-inflammatory cytokine release in response to antigen challenge, possibly coupled with acute suppression of proinflammatory cytokine production, corresponding with studies using mitogen or endotoxin as stimulant. Moderate hypohydration does not appear to influence these changes.

## 1. Introduction

Prolonged, strenuous exercise has been associated with a temporal depression of host defence, increasing susceptibility to opportunistic infections [1, 2]. Almost certainly, this immunosuppression is multifactorial in origin [3, 4]. Reductions in salivary immunoglobulin A secretion [5–7], natural killer cell activity [8], lymphocyte proliferative response [5, 9], and impaired neutrophil phagocytic function [10] following prolonged exercise have been suggested as some of the possible mechanisms and likely explain, at least in part, why elite endurance athletes appear particularly prone to upper respiratory tract infections [11]. Even mild infections that are medically innocuous can significantly disrupt training and impair athletic performance.

Gleeson et al. [12] reported an elevated anti-inflammatory, and specifically interleukin (IL)-10, response to antigen challenge as a risk factor for development of upper respiratory symptoms (URS) in physically active individuals. Furthermore, athletes with a high training load (≥11 h moderate-high intensity training per week) experienced more than twice as many URS episodes and had approximately threefold higher resting IL-2, IL-4, and IL-10 production by antigen-stimulated whole blood culture than athletes with a low (3–6 h per week) training volume [13]. Cytokines play a key, pleiotropic role in orchestrating the responses of lymphocytes, macrophages, and other immune cells during an infection [14–16]. Changes in the profile of cytokine production after acute or chronic exercise may therefore influence infection risk and/or the severity and duration of illness symptoms.

The systemic cytokine response to exercise has been relatively well documented [17, 18]. However, plasma cytokine concentrations in an unstimulated state do not accurately reflect how the body would react in response to a pathogen. As such, these results are of limited relevance when assessing an individual's ability to respond appropriately to an immune challenge. Investigating the cytokine response to stimulation, either* in vitro* or* in vivo*, may provide greater insight as to what extent host defence is compromised following exercise.

Numerous approaches have been employed to study the effect of exercise on the functional capacity of immune cells to produce various cytokines. Although some animal models have used* in vivo* stimulation with specific antigens [19], the majority of studies have used mitogenic plant lectins such as phytohaemagglutinin, phorbol myristate acetate in combination with ionomycin, or endotoxin, principally lipopolysaccharide (LPS), as stimulant, incubated* in vitro* with peripheral blood mononuclear cells (PBMCs) or in whole blood culture. While studies using powerful T-cell mitogens or LPS-stimulation can further our understanding of immunocompetence, in order to elucidate how specific cellular and molecular immune responses to viral and/or bacterial infection are affected by exercise, examining the cytokine response to antigen-stimulation may be more appropriate. Animal models and studies using endotoxin or mitogen stimulation indicate a temporal suppression of the type 1 cytokine response following prolonged exercise, which may be responsible, in part, for the concurrent increase in URS susceptibility. However, to the authors' knowledge, no studies to date have investigated antigen-stimulated cytokine production by human whole blood culture following acute endurance exercise.

The capacity of leukocytes to produce cytokines upon adequate challenge has potentially far reaching consequences for the entire functional capacity of the immune system and is highly likely to reflect the capacity of an individual to defend itself against intruding microorganisms [20]. A model using viral and/or bacterial antigens to stimulate cytokine production in whole blood culture probably comes the closest to the natural environment, avoiding artefacts from cell isolation and preparation and allowing natural interactions between immune components and antigens within the normal hormonal milieu. Essentially it is an* in vitro* method of simulating responses to an infection. The influence of exercise on the cytokine production capacity can be measured by investigating the* in vitro* cytokine response to antigens in blood cell cultures set up before and after exercise. Changes in cytokine production after exercise may arise from both changes in circulating leukocyte subset populations after exercise and changes in the plasma concentrations of stress hormones (e.g., adrenaline and cortisol) and other cytokines (e.g., IL-6 and IL-10).

Studies suggest that athletes frequently commence training or competition in a mildly or moderately hypohydrated state [21–24] and that they typically fail to ingest sufficient fluid to offset losses through sweating during exercise [21, 23, 25, 26]. Compared with exercise in a euhydrated state, moderate dehydration would be expected to result in an augmented stress response and thus likely greater disturbances to immune function. For example, Maresh et al. [27] found that athletes hypohydrated to *∼*5% body mass loss had significantly higher plasma cortisol concentrations prior to and 20 min following acute submaximal exercise. The aims of the current study were therefore to determine (1) the acute effects of a single bout of prolonged exercise on antigen-stimulated cytokine production in whole blood culture and (2) how these effects differ depending on whether exercise is undertaken in a euhydrated or moderately hypohydrated state. It was hypothesised that immediately following 2 h of cycling exercise at 60% $\dot{V}{\text{O}}_{2\max}$, there would be no significant change in antigen-stimulated production of type 2/anti-inflammatory cytokines, but that there would be a transient suppression of type 1/proinflammatory cytokine production relative to baseline, with values returning towards resting levels within 2 h of recovery. In addition, it was hypothesised that exercise in a moderately hypohydrated state would lead to a somewhat augmented cortisol response and greater disturbances of cytokine production compared to exercise in a euhydrated state.

## 2. Materials and Methods

### 2.1. Subjects

Twelve healthy, male university students provided their written, informed consent to participate in the study, which was approved by the Loughborough University ethical advisory committee. Prior to the start of the study, participants completed a health screening questionnaire. Subjects could be included if they were between 18 and 35 years of age, currently healthy and without URS or use of any medication during the past four weeks. Exclusion criteria were smoking, suffering from, or with a history of, cardiac, hepatic, pulmonary, renal, neurological, haematological, psychiatric, or gastrointestinal illness, or haematological values (leukocyte and erythrocyte counts) outside the normal range. Baseline characteristics were as follows (mean ± SD): age 21 ± 1 years, body mass 73.6 ± 7.7 kg, height 1.77 ± 0.07 m, and maximal oxygen uptake ($\dot{V}{\text{O}}_{2\max}$) 57.6 ± 6.1 mL/kg/min.

### 2.2. Laboratory Visits

On the first laboratory visit, 1-2 weeks prior to the first experimental trial, subjects completed a continuous, incremental exercise test to volitional exhaustion on an electromagnetically braked cycle ergometer (Lode Excalibur Sport, Groningen, Netherlands) for determination of $\dot{V}{\text{O}}_{2\max}$. The test began at 95 W with increments of 35 W every 3 min. Rating of perceived exertion (RPE) was noted and expired gas was collected into Douglas bags during the final minute of each stage. Heart rate was measured continuously using short-range telemetry (Polar, Kempele, Finland). A paramagnetic oxygen analyser (Servopro 1440D, Servomex, Crowborough, UK) and infrared carbon dioxide analyser (Servopro 1440D) were used in combination with a dry gas meter (Harvard Apparatus, Edenbridge, UK) for determination of ${\dot{V}}_{E}$, ${\dot{V}\text{O}}_{2}$ and ${\dot{V}\text{CO}}_{2}$. The work rate corresponding to 60% $\dot{V}{\text{O}}_{2\max}$ was then calculated from the ${\dot{V}\text{O}}_{2}$-work rate relationship using a linear equation. After a 15-minute recovery, participants cycled for 20 min at a steady-state work rate equivalent to 60% ${\dot{V}\text{O}}_{2\max}$ with expired gas samples collected after 5, 15, and 20 min in order to familiarize the subjects with the exercise protocol for subsequent trials and to ensure that the calculated work rate elicited the desired relative exercise intensity. The final three days prior to commencing the first experimental trail, participants attended the laboratory in the morning following an overnight fast. On each occasion nude body mass was measured, and the average of these was taken as baseline euhydrated body mass.

For each experimental trial, subjects arrived at the laboratory at 08:00 following an overnight fast of at least 10 h. All trials were completed at the same time of day to reduce intertrial effects of diurnal variations in cytokine production and plasma cortisol [28, 29]. Subjects were requested to refrain from strenuous exercise and, in an effort to standardize their nutritional status, asked to record and replicate as closely as possible their dietary intake during the 24 h leading up to each trial, and instructed to refrain from foods with a high water content. The two trials were conducted in a randomized and counterbalanced order and separated by six or seven days. For the dehydrated trial (DH), fluid intake was restricted to 500 mL of water during the 24 h period prior to the start of the trial, and no fluid was ingested in the morning before or during the exercise test. In the euhydrated trial (EU), subjects were asked to drink normally in the 24 h leading up to the trial and to consume 500 mL of water in the morning 1-2 h before the start of exercise test to ensure adequate hydration. In the EU trial subjects were also provided with 250 mL of water every 20 min during exercise to offset fluid losses through sweating. Before each trial, subjects provided a first pass morning urine sample. The exercise protocol was identical for both trials.

Prior to the start of exercise, subjects voided and nude body mass was recorded. After sitting quietly for 10 min, an initial resting blood sample was obtained. Subjects then cycled for 120 min at 60% $\dot{V}{\text{O}}_{2\max}$ on a stationary cycle ergometer in a laboratory maintained at 21 ± 1°C. Expired gas was collected after 15, 45, 75, and 105 min of exercise to determine ${\dot{V}\text{O}}_{2}$. Heart rate was measured using short-range telemetry (Polar, Finland) at 15-min intervals. Immediately after completion of exercise, blood and urine samples were collected, and body mass measured before participants were provided with 500 mL of water. Subjects then sat quietly for 120 min, before providing a final blood sample. Urine osmolality before and after exercise was determined via freezing-point depression using a single-sample osmometer (Osmomat 030, Gonotec, Berlin, Germany).

### 2.3. Blood Sampling

Blood samples were collected at rest immediately before the start of exercise (t0), immediately after cessation of exercise (t120), and after a 2-h recovery period (t240). On each occasion, a venous blood sample (11 mL) was obtained by venepuncture from an antecubital vein. Blood was collected into two vacutainer tubes (Becton Dickinson, Oxford, UK) containing either lithium heparin or K3EDTA as anticoagulant. Haematological analysis was immediately performed on the K3EDTA sample, as outlined below.

### 2.4. Blood Cell Counts

Blood samples in the K3EDTA vacutainer (4 mL) were used for determination of red blood cell concentration, haematocrit, haemoglobin, and total and differential leukocyte counts using an automated cell-counter (A^c^.T 5diff haematology analyser, Beckman Coulter, High Wycombe, UK). The coefficient of variation for all measured variables was <2.0%. Samples were analysed in duplicate, and the average of both values entered into analyses. Haematocrit and Haemoglobin values were used to calculate changes in plasma volume using the equations of Dill & Costill [30].

### 2.5. Plasma Cortisol

The remaining K3EDTA blood sample was centrifuged for 10 min at 1500 g and 4°C. Plasma was aliquoted and stored at −20°C until analysis. Plasma concentrations of cortisol were determined using a commercially available solid phase competitive enzyme-linked immunosorbent assay (IBL International, Hamburg, Germany) according to the manufacturer's instruction manual, with analytical sensitivity of 2.46 ng*·*mL^−1^ and intra-assay coefficient of variation of <3.0%. Plates were read on a microtitre plate reader at 450 nm. All samples were assayed in duplicate, and the mean of the two readings for each sample was used.

### 2.6. Antigen-Stimulated Cytokine Production

For determination of cytokine production, heparin was used as anticoagulant rather than K3EDTA as EDTA is a calcium chelator and has been found to inhibit cell function and cytokine production in bioassays [31]. Stimulated whole blood culture production of IFN-*γ*, TNF-*α*, IL-1*α*, IL-1*β*, IL-2, IL-4, IL-6, and IL-10 was determined as follows: for each sample, 0.25 mL heparinized whole blood was added to 0.75 mL RPMI medium (Sigma Chemicals, Poole, UK) with no added stimulant (US), or stimulant at a dilution of 1 : 2000 (S). The dilution used was based on a separate experiment (unpublished data), which established the dose–response curve for the measured cytokines over the dilution range of 1 : 200–1 : 20 000. The stimulant used was a commercially available multiantigen (DTaP/IPV/Hib) vaccine containing diphtheria, tetanus, acellular pertussis, poliomyelitis, and Haemophilus influenza type b antigens (Pediacel vaccine, Sanofi Pasteur, Maidenhead, UK). This antigen cocktail was chosen because it is offered routinely on the NHS to all babies in the UK, with the vast majority of individuals being vaccinated against these antigens. Upon subsequent stimulation, a faster and more robust immune response would be expected than if novel antigens were used. All participants in the current study had received the DTaP/IPV/Hib vaccine as infants. The whole blood culture was then incubated for 24 h at 37°C and 5% CO_2_. Following centrifugation for 4 min at 13,400 g in a microcentrifuge, supernatants were collected and stored frozen at −20°C until analysis. Cytokine concentrations were determined with an Evidence Investigator System using the cytokine biochip array EV 3623 (Randox, County Antrim, UK). The intra-assay coefficient of variation was <±5% for all measured cytokines. US values were then subtracted from S values to isolate cytokine production specifically in response to stimulation and account for plasma concentrations and spontaneous production during the 24 h culture period.

### 2.7. Statistical Analysis

Data are presented as Mean ± Standard Deviation. Statistical analyses were performed using IBM SPSS Statistics 19. Changes in total and differential leukocyte counts, plasma cortisol, and antigen-stimulated cytokine production were analysed using a 2-way repeated measures analysis of variance (ANOVA) with trial (DH and EU) and time (before exercise, after exercise, and 2 h after exercise) as factors. Although ANOVA are generally considered robust to minor violations of normality [32], cytokine data that was found to be significantly nonnormal was transformed using a log transformation prior to analysis. The statistical analysis and calculation of summary statistics were subsequently carried out on the transformed data with summary statistics transformed back to the original scale for presentation as geometric means with 95% confidence intervals. A Bonferroni adjustment was used to correct for multiple comparisons. Between-trial differences in mean heart rate, RPE and %*V*O_2max⁡_ during exercise were determined using paired samples *t*-test. Significance was accepted at the *P* < 0.05 level.

## 3. Results

Compared to baseline euhydrated body mass, fluid restriction in the DH trial resulted in body mass loss of 1.3 ± 0.7% and 3.9 ± 1.0% before and after exercise, respectively. Urine osmolality decreased following exercise and was significantly (*P* < 0.01) higher in DH compared to EU at both time points (972 ± 134 versus 707 ± 229 mOsm/kg, and 933 ± 138 versus 555 ± 254 mOsm/kg at pre- and postexercise, resp.). Subjects cycled at an average power output of 175 ± 22 W for the 2 h exercise period. This elicited an exercise ${\dot{V}\text{O}}_{2}$ of 60 ± 4% ${\dot{V}\text{O}}_{2\max}$, which did not differ significantly between trials. Mean heart rate was significantly (*P* < 0.001) lower during exercise in EU than DH (154 ± 10 bpm versus 161 ± 10 bpm). Similarly, RPE was significantly lower for EU than DH (13 ± 2 versus 14 ± 3, *P* < 0.01).

**Figure 1:**
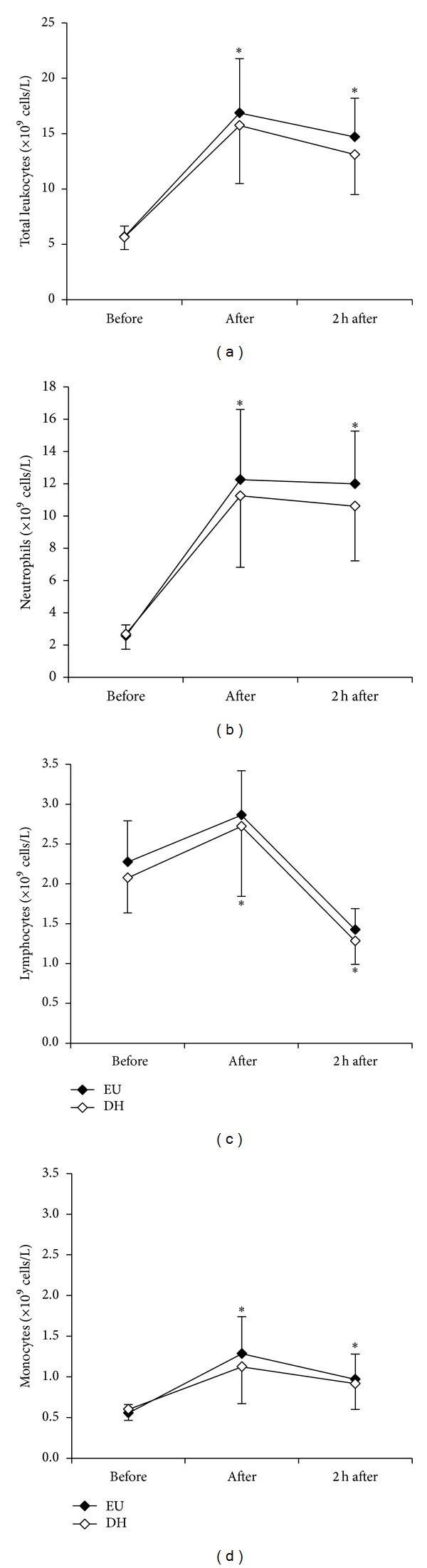
Total leukocyte, neutrophil, lymphocyte, and monocyte counts before, immediately after, and after 2 h recovery following 2 h cycling exercise in euhydrated (EU; filled circles) and dehydrated (DH; open circles) trials. Data are Mean ± SD. *indicates significant difference for both trials from preexercise (*P* < 0.05).

**Figure 2:**
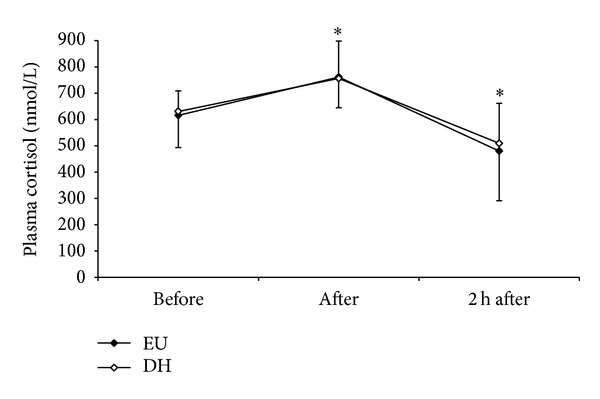
Plasma cortisol concentration before, after, and after 2 h recovery following 2 h cycling exercise in euhydrated (EU; filled circles) and dehydrated (DH; open circles) trials. Data are presented as Mean ± SD. *indicates significant difference for both trials from preexercise (*P* < 0.001).

Red blood cell concentration, haematocrit and haemoglobin concentration were all significantly higher after exercise and at 2 h after exercise. (Table 1). The postexercise increase in haemoglobin was significantly higher in DH than EH. Plasma volume was significantly reduced both immediately after and 2 h after exercise but did not significantly differ between trials, nor did adjusting for plasma volume change the outcomes of statistical tests, and hence the data presented are uncorrected values. Analyses of leukocyte counts demonstrated a significant main effect of time for total and differential leukocytes, with no significant difference between trials. Under both conditions, exercise resulted in a significant increase in total leukocytes, neutrophils, and monocytes above baseline, which persisted during the 2 h period of recovery (Figures 1(a)–1(d)). Lymphocytes also increased significantly immediately following exercise, before dropping below baseline, after 2 h. Plasma cortisol was significantly elevated by approximately 22% (136 nmol/L) postexercise in both trials, with no difference between trials.  Figure 2 shows plasma cortisol concentration before, after, and 2 h after exercise.

**Figure 3:**
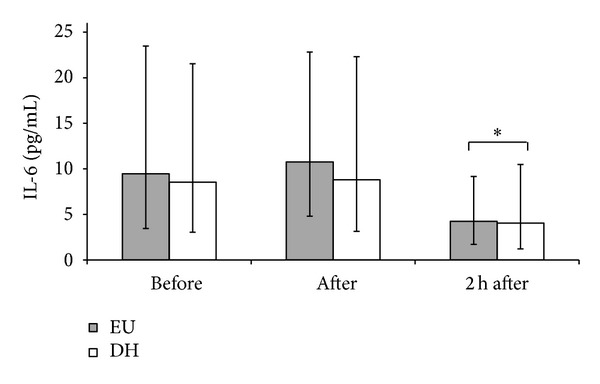
IL-6 production by antigen-stimulated whole blood culture before, after, and 2 h after exercise in euhydrated (EU; filled bars) and dehydrated (DH; open bars). Data are geometric mean with 95% CI. *indicates significant difference for both trials from pre exercise (*P* = 0.01).

Stimulation with antigens resulted in significantly higher levels of all measured cytokines compared to unstimulated samples. Cytokine concentrations following stimulation are presented in Table 2. IL-6 production was significantly (*P* = 0.010) reduced 2 h after exercise (Figure 3). IL-10 production also showed a main effect of time (*P* = 0.023) with higher production immediately after exercise. Although not quite reaching statistical significance, antigen-stimulated IFN-*γ* and IL-2 release tended (0.05 < *P* < 0.10) to decrease following exercise. IL-1*β* and IL-1*α* production were not significantly altered by exercise. No significant effect of hydration status was observed for any of the measured cytokines.

## 4. Discussion

The main findings of the current study are that moderate hypohydration equivalent to ≤4% body mass loss does not appear to influence resting or exercise-induced changes in plasma cortisol, total and differential leukocyte counts, or antigen-stimulated cytokine production. However, they do provide evidence that prolonged exercise may upregulate the production of IL-10 in response to antigen challenge, while inhibiting the production of IL-6 during recovery from a single bout of endurance exercise. Although there was also a consistent trend for suppressed type 1 cytokine production following exercise, due to large individual variations these changes did not quite reach statistical significance.

An enhanced anti-inflammatory response to antigen challenge, as evidenced by potentiated IL-10 production after exercise, indicates a shift in the type 1/type 2 cytokine balance towards type 2. This corresponds with previous studies that have reported exercise-induced changes in T cell number and function resulting in a relatively type 2 dominant cytokine profile [33–36]. However, contrary to the current findings, these studies observed that this shift was primarily due to a reduction in type 1 T cell number and function, with no significant change in type 2 lymphocytes. Although evidence indicates that intracellular expression of IL-4 in both unstimulated and stimulated cultures is increased in certain cell subsets following exercise [37, 38] other studies have found that acute endurance exercise has little or no effect on IL-4 release in response to mitogen stimulation [34, 35], while inducing a temporal suppression of type 1 cytokine production, specifically IFN-*γ* [25, 34, 39–41], IL-2 [40–42], and TNF-*α* [39, 41, 43–46]. To the authors' knowledge, only two previous studies have investigated the acute exercise effects on IL-10 release in humans in response to stimulation. In line with our results, a recent study by LaVoy and colleagues [37] reported significantly increased intracellular expression of IL-10 in CD27-CD8+ T-cells in response to phytohaemagglutinin 1 h after a 40 km cycling time trial. Conversely, Smits et al. [45] reported suppressed* in vitro* IL-10 production in response to LPS following 15–20 min of rowing to volitional exhaustion in competitive oarsmen. Although these results appear contrary to those of the current study, it is difficult to draw direct comparisons due to dissimilarities in the training status of participants, exercise mode, intensity, and duration. Furthermore, the primary cellular source of IL-10 is the lymphocyte, particularly the T regulatory cell subset. LPS-stimulation primarily activates monocytes, with any lymphocyte response being highly monocyte dependent. The pattern of IL-10 release elicited by LPS will therefore likely differ somewhat from that induced by antigen-stimulation. Van der Poll et al. [47] found that prior exposure to adrenaline potentiates LPS-induced IL-10 production in humans, both* in vivo* and* in vitro*, and, alongside the anti-inflammatory effects of elevations in plasma cortisol, may provide a plausible mechanism through which acute exercise augments IL-10 release.

Type 1 cytokines promote cellular immunity by stimulating the functional activity of T cytotoxic cells, NK cells, and activated macrophages [48]. A shift towards a more type 2 dominant cytokine response as seen in the current study will likely compromise cellular immunity while temporarily enhancing humoral immunity, particularly because the two are mutually inhibitory. Dysregulation of the type 1/type 2 cytokine balance has been associated with susceptibility to infectious disease [49]. Viral infection generally leads to a predominantly type 1 cytokine response [50]. Pitkäranta et al. [51] found that lower virus-induced IFN-*γ* production* in vitro* was associated with frequently recurring respiratory infections in children, whereas blocking of Type 2 cytokine expression via prior administration of antibodies to IL-10 and IL-4 reduces symptom severity following respiratory syncytial virus infection [52, 53]. Similarly, URS risk in athletes is associated with increased antigen-stimulated IL-10 production at rest [13]. Hence, a lower type 1/type 2 cytokine ratio in response to immune challenge may compromise host defence against respiratory infections.

Another interesting finding of the current study is that antigen-stimulated IL-6 production was inhibited 2 h after exercise. It is well recognised that IL-6 is released from contracting skeletal muscle resulting in elevated plasma concentrations following exercise [54, 55]. This is in accordance with a significant (*P* < 0.001) exercise-induced increase in IL-6 in unstimulated culture in the current study, with these values primarily reflecting plasma concentrations, plus any spontaneous release during the culture period. However, the results of the current study indicate that the capacity of immune cells to produce IL-6 in response to antigen challenge is inhibited following prolonged exercise. The results of previous studies investigating changes in stimulated IL-6 release following endurance exercise are inconsistent. Smits et al. [45] reported suppressed postexercise IL-6 production in response to* in vitro* stimulation with LPS. Weinstock et al. [41] found that although total LPS-induced IL-6 release appeared unchanged by exercise, when corrected for the number of monocytes in the culture, IL-6 production per cell was significantly lower compared with preexercise. However, Baum et al. [39] found no significant changes in IL-6 production from whole blood cultured with LPS, and Starkie et al. [46] reported no exercise-induced changes in IL-6 production from monocytes, although it is worth noting that the exercise protocol used in that study was not of sufficient intensity to stimulate an increase in plasma cortisol. Haarh et al. [56] reported an increase in IL-6 production 2 h after exercise in response to incubation of PBMCs with LPS. These inconsistencies can likely be explained by variations in exercise protocols and training status of participants, as well as the immunological stimulant, duration of cell culture [57], and culture technique used (e.g., PBMCs versus whole blood culture). Well-regulated cytokine responses are critical for host defence [58], and IL-6 plays a key multifunctional role in orchestrating the acute phase reaction and various immune responses [59], eliciting both pro- and anti-inflammatory effects [60]. The observed exercise-induced inhibition of IL-6, coupled with an enhanced anti-inflammatory cytokine response, may therefore be indicative of somewhat compromised host-defence to viral infection.

It is well-documented that prolonged exercise results in changes in the total and differential numbers of circulating leukocytes along with changes in lymphocyte subsets [61, 62]. Depending on the cytokine and its principal cellular source, shifts in cell numbers will have clear implications for total cytokine production. However, a weakness of the current study was that, since the principal cellular source of IL-10 and IL-6 in antigen-stimulated culture is not known, it was not possible to correct absolute production for such changes. As well as major cell types, shifts in circulating cell (e.g., lymphocyte) subsets with exercise [61] may also have contributed towards changes in total cytokine production, as it is well established that different subsets also vary in their proclivity to secrete different cytokines [63].

The observed changes in cytokine production can likely in part be explained by the exercise-induced elevation in plasma cortisol. A glucocorticoid released from the adrenal cortex in response to stress; cortisol has immunosuppressive and anti-inflammatory effects [64], acting to mediate the recovery from immune activation early in the stress response, thus preventing an “overshoot” of the immune reaction [65]. Cortisol acts to suppress the production of proinflammatory cytokines [29], as well as upregulating the expression of certain anti-inflammatory cytokines [66], thus functioning as a negative feedback mechanism protecting against excessive inflammation. Studies on the effect of elevated plasma cortisol on stimulated cytokine production are somewhat inconsistent. However, elevations in plasma cortisol within the physiological range, such as achieved during strenuous exercise, appear to suppress LPS-induced TNF-*α*, IL-1*β*, and IFN-*γ* production, but interestingly not IL-6 production [29, 67]. In the current study there was no effect of fluid restriction on plasma cortisol. This is somewhat unexpected as heart rate and RPE data suggest that exercise in DH was more physiologically stressful than in the EU condition. Bishop et al. [68] found that fluid ingestion during prolonged exercise attenuated the cortisol response. Mitchell et al. [69], on the other hand, found that although hypohydration resulted in elevated plasma cortisol when exercise was performed in the heat, there was no effect of hydration status on cortisol concentration, total or differential leukocyte numbers, or lymphocyte function when exercising in temperate ambient conditions. The relatively modest level of hypohydration induced in the current study appeared insufficient to influence the plasma cortisol response to exercise at 21°C and subsequently had no significant effect on the measured immune variables. Although it was beyond the scope of the current study to measure multiple hormones, it is worth noting that, in addition to cortisol, several other hormones known to increase with exercise also have immunomodulatory effects, notably adrenaline, noradrenaline, growth hormone, and prolactin [70].

Induction of cytokine production by antigen challenge proceeds with a slower kinetic than that associated with powerful T-cell mitogens used in a number of previous studies and induces lymphocyte responses more directly than LPS challenge. Strength of the current study is that the cytokine response to the multiantigen vaccine used as stimulant can be considered closer to the natural response to such opportunistic infections as may be encountered during the recovery from exercise. It is acknowledged that measuring cytokine production from whole blood culture rather than a known number of PBMCs may be confounded by changes in numbers and proportions of circulating lymphocytes and monocytes with exercise. However, a disadvantage of isolating PBMCs is that this insulates the cells from the influence of other cells, hormones, and cytokines, while whole blood culture more closely replicates the natural milieu. Although changes in leukocyte subsets with exercise will alter the number of cells in whole blood culture and thereby influence total cytokine production, changes in absolute cytokine production in response to an immune challenge are likely to have implications for disease susceptibility, regardless of the underlying mechanism. A weakness of the current study is that very large individual variations in cytokine responses, although perhaps an interesting finding in itself, somewhat limit the interpretation of results. Future studies examining cytokine responses to antigen-challenge may benefit from larger sample sizes in order to improve statistical power.

## 5. Conclusion

Contrary to our hypothesis, moderate hypohydration elicited by a 24 h fluid restriction protocol does not appear to influence plasma cortisol levels, or exacerbate exercise-induced immune disturbances. However, the effects of more severe levels of hypohydration on such immune markers remain unclear. The current study does, however, provide further evidence of a temporal shift towards a more type 2 dominant cytokine response to immune challenge, perhaps indicative of compromised host-defence to viral infection following prolonged exercise. Although the clinical significance of the magnitude of changes observed are not clear, these results may further contribute towards explaining the apparent “open-window” of infection that is evident during short-term recovery from endurance exercise.

## Figures and Tables

**Table 1:** Haematological variables bfore, after, and 2 h after exercise in euhydrated and dehydrated trials.

		Before exercise	After exercise	2 h after exercise
RBC (×10^12^/L)	EU	4.76 (0.35)	4.95 (0.36)*	4.96 (0.44)*
	DH	4.83 (0.40)	5.10 (0.40)*	4.95 (0.48)*
Hct (%)	EU	45.9 (3.2)	47.8 (3.6)*	47.4 (4.4)*
	DH	46.8 (3.0)	49.6 (4.1)*	47.9 (3.5)*
Hb (g/dL)	EU	15.7 (0.7)	16.4 (0.7)*	16.3 (1.1)*
	DH	15.9 (1.0)	16.9 (1.2)^∗†^	16.3 (1.1)*

Data are mean (SD); *indicates significant difference from preexercise (*P* < 0.001); ^†^indicates significant interaction effect (trial ∗ time) (*P* < 0.05).

RBC: red blood cells; Hct: haematocrit; Hb: haemoglobin.

**Table 2:** 24 h cytokine production in response to *in vitro* antigen stimulation of whole blood collected before, after, and 2 h after exercise in euhydrated and dehydrated trials.

	Euhydrated trial			Dehydrated trial			Statistics
	Before exercise	After exercise	2 h after exercise	Before exercise	After exercise	2 h after exercise	
**IL-1** **α** (pg/mL)	**0.40** (0.11–0.76)	**0.22** (0.07–0.38)	**0.25** (0.01–0.56)	**0.27** (0.02–0.56)	**0.22** (0.08–0.38)	**0.19** (0.07–0.32)	
**IL-1** **β** (pg/mL)	**1.80** (0.78–3.42)	**2.50** (1.35–4.21)	**1.81** (1.12–2.71)	**1.95** (0.74–4.02)	**1.73** (0.63–3.59)	**1.38** (0.53–2.69)	
**IL-2** (pg/mL)	**8.01** (2.45–22.5)	**6.93** (2.44–17.3)	**4.80** (1.82–10.9)	**5.56** (1.37–17.2)	**4.31** (1.19–11.9)	**3.23** (1.16–7.30)	
**IL-4** (pg/mL)	**0.65** (0.16–1.14)	**0.73** (0.34–1.11)	**0.86** (0.27–1.44)	**0.58 **(0.25–0.92)	**0.68** (0.32–1.04)	**0.55** (0.31–0.79)	
**IL6** (pg/mL)	**9.45** (3.47–23.5)	**10.8** (4.80–22.8)	**4.24** (1.70–9.16)	**8.54** (3.04–21.5)	**8.81** (3.13–22.3)	**4.05** (1.22–10.47)	**Main effect of time** *F*(1.93) = 5.840, *P* = 0.010 Observed power = 0.801
**IL-10** (pg/mL)	**0.43** (0.09–0.88)	**0.77** (0.57–1.01)	**0.42** (0.16–0.73)	**0.40** (0.11–0.78)	**0.59** (0.34–0.90)	**0.38** (0.15–0.66)	**Main effect of time** *F*(2) = 4.495, *P* = 0.023 Observed power = 0.707
**IFN-** **γ** (pg/mL)	**0.76** (0.18–1.63)	**0.58** (0.17–1.14)	**0.48** (0.02–1.13)	**0.87** (0.26–1.79)	**0.45** (0.05–1.00)	**0.52** (0.16–1.00)	
**TNF-** **α** (pg/mL)	**2.68** (1.04–5.62)	**2.24** (1.38–3.43)	**2.40** (1.17–4.32)	**2.36** (0.77–5.36)	**2.12** (0.78–4.49)	**1.95** (0.80–3.83)	

Data are presented as geometric means (95% CI).

## References

[1] Davis JM, Kohut ML, Colbert LH, Jackson DA, Ghaffar A, Mayer EP (1997). Exercise, alveolar macrophage function, and susceptibility to respiratory infection. *Journal of Applied Physiology*.

[2] Murphy EA, Davis JM, Carmichael MD, Gangemi JD, Ghaffar A, Mayer EP (2008). Exercise stress increases susceptibility to influenza infection. *Brain, Behavior, and Immunity*.

[3] Gleeson M, Bishop NC (2000). Modification of immune responses to exercise by carbohydrate, glutamine and anti-oxidant supplements. *Immunology and Cell Biology*.

[4] Walsh NP, Gleeson M, Shephard RJ (2011). Position statement. Part one: immune function and exercise. *Exercise Immunology Review*.

[5] Nieman DC, Simandle S, Henson DA (1995). Lymphocyte proliferative response to 2.5 hours of running. *International Journal of Sports Medicine*.

[6] Steerenberg PA, van Asperen IA, van Nieuw Amerongen A, Biewenga A, Mol D, Medema GJ (1997). Salivary levels of immunoglobulin A in triathletes. *European Journal of Oral Sciences*.

[7] Tomasi TB, Trudeau FB, Czerwinski D, Erredge S (1982). Immune parameters in athletes before and after strenuous exercise. *Journal of Clinical Immunology*.

[8] Kappel M, Tvede N, Galbo H Evidence that the effect of physical exercise on nk cell-activity is mediated by epinephrine. *Journal of Applied Physiology*.

[9] Henson DA, Nieman DC, Parker JC (1998). Carbohydrate supplementation and the lymphocyte proliferative response to long endurance running. *International Journal of Sports Medicine*.

[10] Müns G (1994). Effect of long-distance running on polymorphonuclear neutrophil phagocytic function of the upper airways. *International Journal of Sports Medicine*.

[11] Boulet LP (2012). Cough and upper airway disorders in elite athletes: a critical review. *British Journal of Sports Medicine*.

[12] Gleeson M, Bishop N, Oliveira M, Tauler P (2013). Influence of training load on upper respiratory tract infection incidence and antigen-stimulated cytokine production. *Scandinavian Journal of Medicine & Science in Sports*.

[13] Gleeson M, Bishop N, Oliveira M, McCauley T, Tauler P, Muhamad AS (2012). Respiratory infection risk in athletes: association with antigen-stimulated IL-10 production and salivary IgA secretion. *Scandinavian Journal of Medicine & Science in Sports*.

[14] Biron CA, Nguyen KB, Pien GC, Cousens LP, Salazar-Mather TP (1999). Natural killer cells in antiviral defense: function and regulation by innate cytokines. *Annual Review of Immunology*.

[15] Samuel CE (2001). Antiviral actions of interferons. *Clinical Microbiology Reviews*.

[16] Trinchieri G (1997). Cytokines acting on or secreted by macrophages during intracellular infection (IL-10, IL-12, IFN-gamma). *Current Opinion in Immunology*.

[17] Ostrowski K, Rohde T, Asp S, Schjerling P, Pedersen BK (1999). Pro- and anti-inflammatory cytokine balance in strenuous exercise in humans. *Journal of Physiology*.

[18] Pedersen BK, Toft AD (2000). Effects of exercise on lymphocytes and cytokines. *British Journal of Sports Medicine*.

[19] Kohut ML, Boehm GW, Moynihan JA (2001). Prolonged exercise suppresses antigen-specific cytokine response to upper respiratory infection. *Journal of Applied Physiology*.

[20] Abbasi A, Fehrenbach E, Hauth M (2013). Changes in spontaneous and LPS-induced *ex vivo* cytokine production and mRNA expression in male and female athletes following prolonged exhaustive exercise. *Exercise Immunology Review*.

[21] da Silva RP, Mündel T, Natali AJ (2012). Pre-game hydration status, sweat loss, and fluid intake in elite Brazilian young male soccer players during competition. *Journal of Sports Sciences*.

[22] Aragon-Vargas LF, Moncada-Jimenez J, Hernandez-Elizondo J, Barrenechea A, Monge-Alvarado M (2009). Evaluation of pre-game hydration status, heat stress, and fluid balance during professional soccer competition in the heat. *European Journal of Sport Science*.

[23] Maughan RJ, Shirreffs SM, Merson SJ, Horswill CA (2005). Fluid and electrolyte balance in elite male football (soccer) players training in a cool environment. *Journal of Sports Sciences*.

[24] Volpe SL, Poule KA, Bland EG (2009). Estimation of prepractice hydration status of National Collegiate Athletic Association Division I athletes. *Journal of Athletic Training*.

[25] Beis LY, Wright-Whyte M, Fudge B, Noakes T, Pitsiladis YP (2012). Drinking behaviors of elite male runners during marathon competition. *Clinical Journal of Sport Medicine*.

[26] Maughan RJ, Merson SJ, Broad NP, Shirreffs SM (2004). Fluid and electrolyte intake and loss in elite soccer players during training. *International Journal of Sport Nutrition and Exercise Metabolism*.

[27] Maresh CM, Whittlesey MJ, Armstrong LE (2006). Effect of hydration state on testosterone and cortisol responses to training-intensity exercise in collegiate runners. *International Journal of Sports Medicine*.

[28] Kanaley JA, Weltman JY, Pieper KS, Weltman A, Hartman ML (2001). Cortisol and growth hormone responses to exercise at different times of day. *Journal of Clinical Endocrinology & Metabolism*.

[29] Petrovsky N, McNair P, Harrison LC (1998). Diurnal rhythms of pro-inflammatory cytokines: regulation by plasma cortisol and therapeutic implications. *Cytokine*.

[30] Dill DB, Costill DL (1974). Calculation of percentage changes in volumes of blood, plasma, and red-cells in dehydration. *Journal of Applied Physiology*.

[31] Cannon JG, Nerad JL, Poutsiaka DD, Dinarello CA (1993). Measuring circulating cytokines. *Journal of Applied Physiology*.

[32] Maxwell SE, Delaney HD (1990). *Designing Experiments and Analyzing Data: Model Comparison Perspective*.

[33] Ibfelt T, Petersen EW, Bruunsgaard H, Sandmand M, Pedersen BK (2002). Exercise-induced change in type 1 cytokine-producing CD8^+^ T cells is related to a decrease in memory T cells. *Journal of Applied Physiology*.

[34] Lancaster GI, Halson SL, Khan Q (2004). Effects of acute exhaustive exercise and chronic exercise training on type 1 and type 2 T lymphocytes. *Exercise Immunology Review*.

[35] Lancaster GI, Khan Q, Drysdale PT (2005). Effect of prolonged exercise and carbohydrate ingestion on type 1 and type 2 T lymphocyte distribution and intracellular cytokine production in humans. *Journal of Applied Physiology*.

[36] Steensberg A, Toft AD, Bruunsgaard H, Sandmand M, Halkjær-Kristensen J, Pedersen BK (2001). Strenuous exercise decreases the percentage of type 1 T cells in the circulation. *Journal of Applied Physiology*.

[37] LaVoy EC, Bosch JA, Lowder TW, Simpson RJ (2013). Acute aerobic exercise in humans increases cytokine expression in CD27^−^ but not CD27^+^ CD8^+^ T-cells. *Brain Behavior and Immunity*.

[38] Zaldivar F, Wang-Rodriguez J, Nemet D (2006). Constitutive pro- and anti-inflammatory cytokine and growth factor response to exercise in leukocytes. *Journal of Applied Physiology*.

[39] Baum M, Müller-Steinhardt M, Liesen H, Kirchner H (1997). Moderate and exhaustive endurance exercise influences the interferon-*γ* levels in whole-blood culture supernatants. *European Journal of Applied Physiology and Occupational Physiology*.

[40] Starkie RL, Rolland J, Febbraio MA (2001). Effect of adrenergic blockade on lymphocyte cytokine production at rest and during exercise. *American Journal of Physiology*.

[41] Weinstock C, König D, Harnischmacher R, Keul J, Berg A, Northoff H (1997). Effect of exhaustive exercise stress on the cytokine response. *Medicine and Science in Sports and Exercise*.

[42] Lewicki R, Tchorzewski H, Majewska E, Nowak Z, Baj Z (1988). Effect of maximal physical exercise on T-lymphocyte subpopulations and on interleukin 1 (IL 1) and interleukin 2 (IL 2) production *in vitro*. *International Journal of Sports Medicine*.

[43] Drenth JPH, van Uum SHM, van Deuren M, Pesman GJ, van der Ven-Jongekrijg J, van der Meer JWM (1995). Endurance run increases circulating IL-6 and IL-1ra but downregulates *ex vivo* TNF-*α* and IL-1*β* production. *Journal of Applied Physiology*.

[44] Kveramo H, Olsen JO, Osterud B (1992). Changes in blood cell response following strenuous physical exercise. *European Journal of Applied Physiology and Occupational Physiology*.

[45] Smits HH, Grünberg K, Derijk RH, Sterk PJ, Hiemstra PS (1998). Cytokine release and its modulation by dexamethasone in whole blood following exercise. *Clinical and Experimental Immunology*.

[46] Starkie RL, Angus DJ, Rolland J, Hargreaves M, Febbraio MA (2000). Effect of prolonged, submaximal exercise and carbohydrate ingestion on monocyte intracellular cytokine production in humans. *Journal of Physiology*.

[47] van der Poll T, Coyle SM, Barbosa K, Braxton CC, Lowry SF (1996). Epinephrine inhibits tumor necrosis factor-*α* and potentiates interleukin 10 production during human endotoxemia. *Journal of Clinical Investigation*.

[48] Gleeson M, Bishop NC, Walsh NP (2013). *Exercise Immunology. Textbook for Undergraduates*.

[49] Lucey DR, Clerici M, Shearer GM (1996). Type 1, and Type 2 cytokine dysregulation in human infectious, neoplastic, and inflammatory diseases. *Clinical Microbiology Reviews*.

[50] Mosmann TR, Coffman RL (1989). TH1 and TH2 cells: different patterns of lymphokine secretion lead to different functional properties. *Annual Review of Immunology*.

[51] Pitkäranta A, Nokso-Koivisto J, Jäntti V, Takala A, Kilpi T, Hovi T (1999). Lowered yields of virus-induced interferon production in leukocyte cultures and risk of recurrent respiratory infections in children. *Journal of Clinical Virology*.

[52] Connors M, Giese NA, Kulkarni AB, Firestone C-Y, Morse HC, Murphy BR (1994). Enhanced pulmonary histopathology induced by Respiratory Syncytial Virus (RSV) challenge of formalin-inactivated RSV-immunized BALB/c mice is abrogated by depletion of interleukin-4 (IL-4) and IL-10. *Journal of Virology*.

[53] Tang Y-W, Graham BS (1994). Anti-IL-4 treatment at immunization modulates cytokine expression, reduces illness, and increases cytotoxic T lymphocyte activity in mice challenged with respiratory syncytial virus. *Journal of Clinical Investigation*.

[54] Ostrowski K, Rohde T, Zacho M, Asp S, Pedersen BK (1998). Evidence that interleukin-6 is produced in human skeletal muscle during prolonged running. *Journal of Physiology*.

[55] Steensberg A, van Hall G, Osada T, Sacchetti M, Saltin B, Pedersen BK (2000). Production of interleukin-6 in contracting human skeletal muscles can account for the exercise-induced increase in plasma interleukin-6. *Journal of Physiology*.

[56] Haahr PM, Pedersen BK, Fomsgaard A (1991). Effect of physical exercise on *in vitro* production of interleukin 1, interleukin 6, tumour necrosis factor-*α*, interleukin 2 and interferon-*γ*. *International Journal of Sports Medicine*.

[57] Reddy M, Eirikis E, Davis C, Davis HM, Prabhakar U (2004). Comparative analysis of lymphocyte activation marker expression and cytokine secretion profile in stimulated human peripheral blood mononuclear cell cultures: an *in vitro* model to monitor cellular immune function. *Journal of Immunological Methods*.

[58] Cross A, Asher L, Seguin M (1995). The importance of a lipopolysaccharide-initiated, cytokine-mediated host defense mechanism in mice against extraintestinally invasive *Escherichia coli*. *Journal of Clinical Investigation*.

[59] Kopf M, Baumann H, Freer G (1994). Impaired immune and acute-phase responses in interleukin-6-deficient mice. *Nature*.

[60] Xing Z, Gauldie J, Cox G (1998). IL-6 is an antiinflammatory cytokine required for controlling local or systemic acute inflammatory responses. *Journal of Clinical Investigation*.

[61] Ricken K-H, Rieder T, Hauck G, Kindermann W (1990). Changes in lymphocyte subpopulations after prolonged exercise. *International Journal of Sports Medicine*.

[62] Shek PN, Sabiston BH, Buguet A, Radomski MW (1995). Strenuous exercise and immunological changes: a multiple-time-point analysis of leukocyte subsets, CD4/CD8 ratio, immunoglobulin production and NK cell response. *International Journal of Sports Medicine*.

[63] Street NE, Mosmann TR (1991). Functional diversity of T lymphocytes due to secretion of different cytokine patterns. *FASEB Journal*.

[64] Auphan N, DiDonato JA, Rosette C, Helmberg A, Karin M (1995). Immunosuppression by glucocorticoids: inhibition of NF-*κ*B activity through induction of I*κ*B synthesis. *Science*.

[65] Sorrells SF, Sapolsky RM (2007). An inflammatory review of glucocorticoid actions in the CNS. *Brain, Behavior, and Immunity*.

[66] Elenkov IJ, Chrousos GP (2002). Stress hormones, proinflammatory and antiinflammatory cytokines, and autoimmunity. *Annals of the New York Academy of Sciences*.

[67] DeRijk R, Michelson D, Karp B (1997). Exercise and circadian rhythm-induced variations in plasma cortisol differentially regulate interleukin-1*β* (IL-1*β*), IL-6, and tumor necrosis factor-*α* (TNF*α*) production in humans: high sensitivity of TNF*α* and resistance of IL-6. *Journal of Clinical Endocrinology & Metabolism*.

[68] Bishop NC, Scanlon GA, Walsh NP, McCallum LJ, Walker GJ (2004). No effect of fluid intake on neutrophil responses to prolonged cycling. *Journal of Sports Sciences*.

[69] Mitchell JB, Dugas JP, McFarlin BK, Nelson MJ (2002). Effect of exercise, heat stress, and hydration on immune cell number and function. *Medicine and Science in Sports and Exercise*.

[70] Pedersen BK, Hoffman-Goetz L (2000). Exercise and the immune system: regulation, integration, and adaptation. *Physiological Reviews*.

